# Mitigation of biogenic methanethiol using bacteriophages in synthetic wastewater augmented with *Pseudomonas putida*

**DOI:** 10.1038/s41598-023-46938-8

**Published:** 2023-11-09

**Authors:** Niti Sarat, Amrita Salim, Sanjay Pal, Suja Subhash, Megha Prasad, Bipin G. Nair, Ajith Madhavan

**Affiliations:** School of Biotechnology, Amrita Vishwa Vidyapeetham, Clappana, Kerala 690525 India

**Keywords:** Biotechnology, Microbiology, Environmental sciences

## Abstract

Wastewater malodour is the proverbial ‘elephant in the room’ notwithstanding its severe implications on sanitation, health, and hygiene. The predominant malodorous compounds associated with wastewater treatment plants and toilets are volatile organic compounds, such as hydrogen sulphide, ammonia, methanethiol, and organic acids. Among them, methanethiol warrants more attention owing to its relatively low olfactory threshold and associated cytotoxicity. This requires an efficient odour-abatement method since conventional techniques are either cost-prohibitive or leave recalcitrant byproducts. Bacteriophage-based methodology holds promise, and the described work explores the potential. In this study, a non-lysogenous *Pseudomonas putida* strain is used as a model organism that produces methanethiol in the presence of methionine. Two double-stranded DNA phages of genome sizes > 10 Kb were isolated from sewage. ɸPh_PP01 and ɸPh_PP02 were stable at suboptimal pH, temperature, and at 10% chloroform. Moreover, they showed adsorption efficiencies of 53% and 89% in 12 min and burst sizes of 507 ± 187 and 105 ± 7 virions per cell, respectively. In augmented synthetic wastewater, ɸPh_PP01 and ɸPh_PP02 reduced methanethiol production by 52% and 47%, respectively, with the concomitant reduction in *P. putida* by 3 logs in 6 h. On extension of the study in *P. putida* spiked-sewage sample, maximum reduction in methanethiol production was achieved in 3 h, with 49% and 48% for ɸPh_PP01 and ɸPh_PP02, respectively. But at 6 h, efficiency reduced to 36% with both the phages. The study clearly demonstrates the potential of phages as biocontrol agents in the reduction of malodour in wastewater.

## Introduction

Close to 50 percentage of domestic wastewater in India is handled by decentralised treatment systems such as septic tanks and pit latrines. Even though they are suitable, cost-effective, and efficient, they suffer from inadequate disinfection and emanation of malodour^1^. The nagging issue of non-compliance with toilet usage is predominately associated with malodour apart from other socio-economic drivers. Hence, malodour, is also remotely linked to open defecation and associated negative health implications^2^. In a decentralised system, both the front end of the toilets, which includes the commode, and the back end constituting the treatment systems, contribute to the biogenesis of malodour^3–5^. The key malodourants are hydrogen sulphide (H_2_S), methanethiol (CH_3_SH), ammonia (NH_3_), and organic acids. Among them, methanethiol is strongly associated with wastewater malodour when compared to other sulphur-containing compounds and organic acids, owing to its low odour threshold (0.0014 ppb), and hence demands increased attention^6–8^. Also referred to as methyl mercaptan, methanethiol is a degradation product of organosulphur compounds and produces a characteristic putrid smell akin to decaying cabbage^9^. Different microorganisms, including *Pseudomonas*, *Serratia*, and *Proteus*, are implicated in its generation^10^. In aerobic environments such as terrestrial soils, several bacteria from the genera *Bacillus* sp, *Arthrobacter* sp, and *Delftia* sp were also reported to produce methanethiol with methionine as the sole carbon source^11^. Microorganisms generate methanethiol via different pathways, broadly classified as assimilatory, aerobic, and anaerobic. In *P. putida,* methionine is desulphurised to methanethiol by methionine gamma-lyase through an assimilatory pathway^6^. On the other hand, marine phytoplankton, through a modified assimilatory mechanism, produces dimethylsulfoniopropionate (DMSP), which is further degraded to methanethiol^12^. In *Hyphomicrobium* sp, it is produced through the oxidation of dimethyl sulphide (DMS) catalysed by the enzyme methanethiol oxidase (MTO)^13^. Sulphide-rich soil is more conducive to aerobic biogenesis than the ferruginous environment^14^. Alternatively, anaerobic biogenesis involves the transfer of methyl groups from S-adenosylmethionine to sulphide, catalysed by thiol S-methyltransferases^15^. It is also important to note that the pathways involved are greatly influenced by the chemical nature of the environment, such as pH, redox potential, the concentration of metal ions, etc^16^. Methanethiol, apart from being a malodourant, is also implicated in cytotoxic effects on mammalian cells, leading to necrosis and neurotoxicity^17,18^. It also indirectly causes corrosion of concrete structures through its microbial conversion to sulfuric acid^19^. As a fallout, around 20–50 billion US dollars are spent annually for the replacement and rehabilitation of sewer pipes in countries like Germany, the US, and Australia^20^.

Treatment strategies for malodour reduction involve biological and chemical scrubbing, biofiltration, the use of biotrickling filters, and adsorption techniques^21^. However, these methods suffer from disadvantages such as high operating costs and the generation of secondary pollutants, namely, ozone and chlorinated byproducts^22–24^. The inefficiency of conventional technologies to remove volatile compounds like methanethiol warrants alternative biological treatment that is cost-effective and eco-friendly. Bacteriophages, being obligate parasites of bacteria, could be exploited for such a task since they are specific and only replicate in the presence of their bacterial hosts ^25^. Bacteriophages are the most abundant entities on the planet, with an estimated 10^31^ viral particles. Broadly based on their life cycle, phages are divided into two groups viz., lytic and temperate (can carry out both lytic and lysogenic cycle). The lytic cycle culminates in the lysis of the host and the release of a crop of phages; on the contrary, the lysogenic cycle yield virocells- hosts with incorporated phage genomes (prophage) in their chromosomes. Temperate phages have the wherewithal to operate both cycles^26^. Environmental applications, such as malodour removal from wastewater, prefer lytic phages over their temperate counterparts for their efficiency and handicap to transfer virulence and antibiotic resistance genes^27,28^. Phage morphology also plays a significant role in the infection, with tailed phages being more efficient, as demonstrated indirectly by their predominance in different environments^29^. Previous work from this group has used phages in reducing H_2_S in synthetic sewage and real samples^30^. Though the potential is well established in clinical, veterinary, and agricultural settings, their application in wastewater remains grossly underexplored, barring a few applications, such as its usage in controlling antifoaming in wastewater treatment plants and disinfection^31–33^. Lysates with phages in isolation or as cocktails can be formulated to target bacterial species. Phages as a biocontrol agent have several advantages over other wastewater treatment methods, such as efficiency and sustainability due to the inherent auto-dosing, and are non-toxic to the environment since being target specific. Moreover, they can efficiently infect recalcitrant bacteria such as cyanobacteria, filamentous bacteria, and biofilm-forming bacteria^34–36^. The described study employed a non-lysogenous, methanethiol-producing strain of *P. putida* as the model organism. Two lytic phages, viz. ɸPh_PP01 and ɸPh_PP02 specific to the model organism, were isolated from sewage and were characterised with regard to pH, temperature, chloroform tolerance, restriction profile, adsorption efficiency, and one-step growth curve. Proof of concept (POC) of reduction in methanethiol biogenesis using these phages was established in synthetic and real sewage.

In brief, the goal of the study is to establish the prospective nature of the proposed bacteriophage-based strategy to reduce the biogenesis of methanethiol. Characterised phages will be employed against established methanethiol-producing bacterial strain propagated in synthetic, and real sewage.

## Materials and methods

### Bacterial strains

*P. putida* 21DW (Accession No: OP808212) was isolated from the roots of *Lemna minor* L (common duckweed) sourced from the vicinity of Amrita Vishwa Vidyapeetham campus, Amritapuri, Kerala, India. The strain was cultured in Luria Bertani broth (HiMedia Laboratories, India) supplemented with 10 mM L-methionine (Sigma-Aldrich, USA)^37^. Other bacterial strains used in the study are listed in Table 1. The voucher specimen was identified by Dr. Kiranraj M S, Assistant Professor, Sree Narayana College, Kerala, India, and was deposited in Sree Narayana College Herbarium (Accession code: SNCH 5002).

**Table 1:** List of bacterial strains used for establishing host range specificity.

Bacteria	Strain designation
*E. coli*	ST155
*Pseudomonas fluorescence*	MTCC 1749
*Pseudomonas aeruginosa*	MTCC PAO1
*Klebsiella pneumoniae*	MTCC 3384
*Salmonella enterica*	MW116733
*E. coli*	MDR
*Proteus vulgaris*	MTCC 7299
*Serratia marcesens*	MTCC 97
*Acinetobacter baumannii*	MTCC 1425
*Staphylococcus aureus*	MRSA

### Antibiogram of the host strain

Antibiotic sensitivity assay was carried out with representatives from different classes of antibiotics (HiMedia Laboratories, India) viz. β-lactams (Ticarcillin-75 mcg), 3^rd^ generation cephalosporins (Ceftazidime-10 mcg), monobactams (Aztreonam-30 mcg), carbapenems (Imipenem-10 mcg), aminoglycosides (Amikacin-30 mcg), fluoroquinolones (Ciprofloxacin-5 mcg), using Kirby-Bauer disk diffusion method. Individual bacterial cultures (3 × 10^8^ CFU/mL) were swabbed onto MHA agar plates and incubated at 37 ℃ overnight. The zone of inhibition (mm) was measured and interpreted as per the EUCAST guidelines^38^.

### Bacteriophage isolation

Wastewater samples were collected from the pilot scale, vertical garden-based, municipal wastewater treatment facility of Amrita Vishwa Vidyapeetham, Amritapuri campus, Kerala, India. 22.5 mL of the sample was added to 2.5 mL of deca-strength bacteriophage broth (peptone—100 g/L, yeast extract powder—50 g/L, NaCl—25 g/L, K_2_HPO_4_− 80 g/L) with 500 µL of *P. putida* culture 0.4 OD_600_ and enriched overnight at 37 ℃ at 200 rpm^39^. After overnight incubation, the enriched broth was centrifuged at 7000×*g* for 15 min at 4 ℃, and the lysate was prepared by passing the supernatant through a 0.22 µm membrane filter (Cole-Parmer, PES membrane, 25 mm). The presence of *P. putida* phages in the crude lysate was checked by a spot assay. 10 µL of the sample was spotted onto the LB agar plate (priorly swabbed with bacterial culture) and incubated overnight at 37 ℃; the zone of clearance indicated the presence of phages in the sample.

### Phage purification and TEM analysis

Heterogeneity of the lysate was initially checked with the agar overlay method, in which 100 µL of the crude lysate was serially diluted in 900 µL of SM buffer (NaCl—5.8 g/L, MgSO_4_− 2 g/L, 1 M Tris–HCl—50 mL/L, pH 7.4) up to 10^–8^ dilutions, 100 µL from each of the dilutions and 100 µL of *P. putida* culture were then mixed with 0.7% soft agar and poured on to LB agar plates (2% agar), and incubated at 37 ℃ overnight. Morphologically different plaques were picked and suspended in 100 µL of SM buffer. The process was repeated five times in order to obtain a pure single phage lysate. Further, the titre of the isolated phages was determined by spot assay and expressed in PFU/mL^40^.

The phage stocks were maintained at 4 ℃ until further use, and glycerol stocks of the phages were maintained at − 80 ℃ for long-term storage. TEM analysis of the phages at high titre was done using FEI Tecnai G2 Spirit Bio Twin at 120 kV at the Central Instrumentation Facility, Indian Institute of Science Education and Research (IISER-TVM), Trivandrum, Kerala, India. Dimensions of the phages were deduced from the micrograph using ImageJ software.

### Phage host range (tropism) determination

Spot assay was employed to assess the host range of the phages against nine Gram-negative and one Gram-positive clinically important bacterial strains (Table 1)^41^. 5 µL of the phage samples were spotted on the surface of the LB agar plates, priorly swabbed with respective bacterial culture at 1 OD_600_. After incubation, at 37 ℃ for overnight, the plates were observed for clearance.

### Phage adsorption assay

The adsorption rate of phages was determined through the protocol described by Kim et al. ^42^. 4 mL of *P. putida* culture (3 × 10^8^ CFU/mL) was mixed with 1 mL of phages (10^7^ PFU/mL), thereby achieving a multiplicity of infection (MOI) of 0.1 and was allowed to adsorb at 37 ℃. 300 µL of the suspension was withdrawn every 2 min up to 12 min and centrifuged at 10000×*g* for 10 min at 4 ℃. The titre of the unadsorbed phages in the supernatant was determined by a double agar overlay method.

### One-step growth Curve

A one-step growth curve experiment was performed with the method described by Jiangtao^43^. *P. putida* was inoculated in LB broth and incubated at 37 ℃ at 200 rpm until 0.2–0.3 OD_600_ was reached. Then, 1 mL of the culture was pelleted down at 13,000×*g* for 5 min at ambient temperature (28 ℃). The pellet was washed three times and resuspended in 900 µL of SM buffer. The suspension (10^7^ CFU/mL) was then mixed with 100 µL of phages (10^6^ PFU/mL) in order to achieve an MOI of 0.1 and incubated at 37 ℃ for 15 min for adsorption. Following which, the sample was centrifuged at 13,000×*g* for 5 min at ambient temperature. The pellet was resuspended in 1 mL of LB broth, diluted to 10^–4^ in 10 mL LB broth, and incubated at 37 ℃ at 200 rpm. Aliquots of 100 µL were removed every 10 min up to 100 min and mixed with 100 µL bacterial culture and 5 mL of 0.7% soft agar and plated by double agar overlay method. Burst size was calculated as the ratio of the average titre of phages in the latent period to the average titre released after the burst.

### pH, temperature, and chloroform stability

The phage stability was assessed by incubating the isolated phages in SM buffer at different pH (2, 4, 6, 7, 10, respectively) at 37 ℃ for 1 h, followed by a spot assay to assess its viability. The stability of phages under different temperatures was also evaluated by incubating them for 1 h at 4 ℃, 20 ℃, 37 ℃ and 50 ℃. After bringing it to room temperature, titre of the phage lysates was determined by spot assay against *P. putida*^44^. To evaluate the effect of chloroform, phage lysates were treated with 10% chloroform, gently mixed, and incubated at 37 ℃ for 1 h with intermittent shaking. The phage titre was determined by spot assay^45^.

### Phage DNA extraction

The genomic DNA extraction of phages was carried out by the phenol–chloroform method^46^. 1 mL of 20% PEG (PEG 8000- 200 g/L, 2.5 M NaCl- 146 g/L) precipitated phage lysate was centrifuged at 18,000×*g* for 30 min at 4 ℃ and resuspended in 500 µL of 5 mM MgSO_4_. The samples were treated with 1.25 µL of DNase I (1000 U, Thermo Fisher Scientific, Lithuania) and Rnase I (10 mg/mL, Thermo Fisher Scientific, Lithuania) at 37 ℃ for 1 h. After incubation, 1.2 µL of Proteinase K (Thermo Fisher Scientific, Lithuania), 20 µL of 0.5 M EDTA, and 25 µL of 10% SDS were added and kept for incubation at 60 ℃ for 1 h. After incubation, samples were allowed to cool at room temperature and centrifuged at 3000×*g* for 5 min at ambient temperature after the addition of an equal volume of phenol: chloroform (1:1). The upper aqueous phase was transferred carefully, and an equal volume of chloroform was added, and centrifuged as previously described. After transferring the aqueous layer, 50 µL of 3 M sodium acetate (NaOAc⋅3H_2_O- 40.8 g/L, pH 4.8) and 2.5 volumes of ice-cold ethanol were added, and the sample was incubated at – 20 ℃ overnight to precipitate the DNA. After incubation, the samples were centrifuged at 3000×*g* for 5 min, and the pellet was resuspended in 50 µL of molecular-grade water. The size and quality of the DNA sample were estimated by running the samples in 1% agarose (HiMedia Laboratories, India) gel electrophoresis, and the DNA was quantified using a Nanodrop spectrophotometer (Thermo Fisher Nanodrop 2000).

### Restriction digestion

The isolated phage DNA was digested with restriction enzymes *Eco*RI, *Bam*HI, *Hind*III (Takara, Japan), *Hpa*I, and *Cla*I (Thermo Fisher Scientific, Lithuania), as per the manufacturer’s recommendations. The digested DNA fragments were separated by gel electrophoresis in 1% agarose and visualized in a gel documentation system (BioRad, USA).

### Bacteriolytic activity of phages at different MOI

The activity of phages on *P. putida* was determined by a bacteriolytic assay in a 96-well plate^47^. 100 µL of 10^8^ CFU/mL of the host and 100 µL of phage suspensions at different MOI of 0.1, 1,10, and 100 were added, mixed, and incubated at 37 ℃ in a rotary shaker at 200 rpm. OD_600_ was taken every 1 h up to 5 h to evaluate the bactericidal activity of phages. Bacterial suspension without phages was used as a control.

### Bacteriophage Insensitive Mutants (BIM) frequency

BIM frequency of the host bacteria was investigated against the isolated phages according to a modified protocol^48^. 100 µL of the bacteria at 0.3 OD_600_ (10^8^ CFU/mL) was mixed with 100 µL of phage suspension with a titre of 10^9^ PFU/mL at an MOI of 10 and incubated for 15 min at 37 ℃ to allow phage adsorption. After incubation, plaque assay was done, and the plates were incubated at 37 ℃. All the colonies that appeared within phage clearance after 24 h were considered as potential BIMs. Phage resistance was further confirmed by spot assay.

$${\text{BIM frequency }}\left( {\text{CFU/mL/24 h}} \right) = \frac{{\text{Phage - resistant mutants in CFU/mL}}}{{\text{Phage - sensitive wild type in CFU/mL}}}$$

### Mitomycin C induction

To check whether *P. putida* contained prophages, mitomycin C induction was performed as described previously^49^. 200 µL of bacterial culture of 0.2 OD_600_ was induced with different concentrations (0.5, 1.3, and 3 µg/mL) of mitomycin C (10 mg, Merck, USA), and culture devoid of mitomycin C served as control. After incubation at 37 ℃, bacterial growth was measured at 0, 1.5, 3, 8, and 24 h. Finally, in order to check prophage induction, the cells were centrifuged at 7000×*g* for 2 min after 24 h incubation and checked for the presence of phages by spot assay.

### Analysis of the reduction of biogenic methanethiol by phages using Ellman’s assay

A phage-mediated reduction of biogenic methanethiol production by *P. putida* was measured using Ellman’s assay. Bacteria were acclimatised to synthetic sewage (peptone—160 mg/L, meat extract—110 mg/L, urea—30 mg/L, K_2_HPO_4_—28 mg/L, NaCl—7 mg/L, CaCl_2_—4 mg/L, MgSO_4_.2H_2_O—2 mg/L) with 10 mM L-methionine as the sulphur donor. The experiment was performed in 24 well plate, with each well containing 800 µL of 10^7^ CFU/mL *P. putida*, to which 400 µL 10 mM 5,5′-dithiobis-(2-nitrobenzoic acid) (DTNB) was added along with 100 µL of phages (10^9^ PFU/mL). Bacterial culture along with 100 µL of SM buffer served as control. The samples were thoroughly mixed, sealed with parafilm, and kept for incubation at 37 ℃ in the dark. The formation of yellow-coloured 2-nitro-5-thiobenzoic acid (TNB) was read at 412 nm at different time points viz. 0 h, 3 h, and 6 h. A linear standard curve was plotted with cysteine hydrochloride monohydrate (H-Cys-OH.HCl.H_2_O) as standard at concentrations ranging from 0 to 1.25 mM (Supplementary file, Fig. S3) for the quantification of methanethiol^50^ . The concomitant reduction in bacterial load was also checked by the plate count method. The assay involves centrifugation of the suspension at 7000×*g* for 5 min and diluted in 0.85% saline, and each of the dilutions was spotted on LB agar plates and incubated at 37 ℃ overnight. Reduction in bacterial count was expressed in CFU/mL.

### Phage treatment on *P. putida* spiked sewage

The potential of phages to reduce methanethiol was assessed in real wastewater using Ellman’s assay. The sample (sourced from the university premises) was centrifuged at 7000×*g* for 5 min to remove any large particulate matter and spiked with *P. putida* at 10^6^ CFU/mL containing 10 mM methionine. The experiment was performed in 24 well plate, with each well containing 800 µL of 10^6^ CFU/mL *P. putida*, to which 400 µL 20 mM DTNB was added along with 100 µL of phages (10^8^ PFU/mL). Bacterial culture along with 100 µL of SM buffer served as control. The assay methodology is the same as described previously. The methanethiol reduction efficiency was expressed in percentage.

### Statistical analysis

GraphPad Prism version 8.0.2 (GraphPad Software, Inc, San Diego, CA) was used to perform all the statistical analysis. pH stability analysis was performed using an ordinary 2-way ANOVA, temperature and chloroform stability studies were analysed using two-way ANOVA followed by Sidak’s test, the bacterial killing assay was performed using two-way ANOVA followed by Tukey’s test, and prophage induction studies by two-way ANOVA followed by Dunnett’s test. Cell count reduction was analysed using one-way ANOVA followed by Dunnett’s multiple comparison test with a single pooled variance. A value of p ≤ 0.05 was considered statistically significant.

### Statement of compliance

Experimental research on *Lemna minor* L described here complies with relevant institutional, national and international guidelines and legislation. Moreover, the species belongs to the IUCN ‘Least concern’ category.

## Results

### Characterisation of phage isolates

#### Morphology

**Figure 1:**
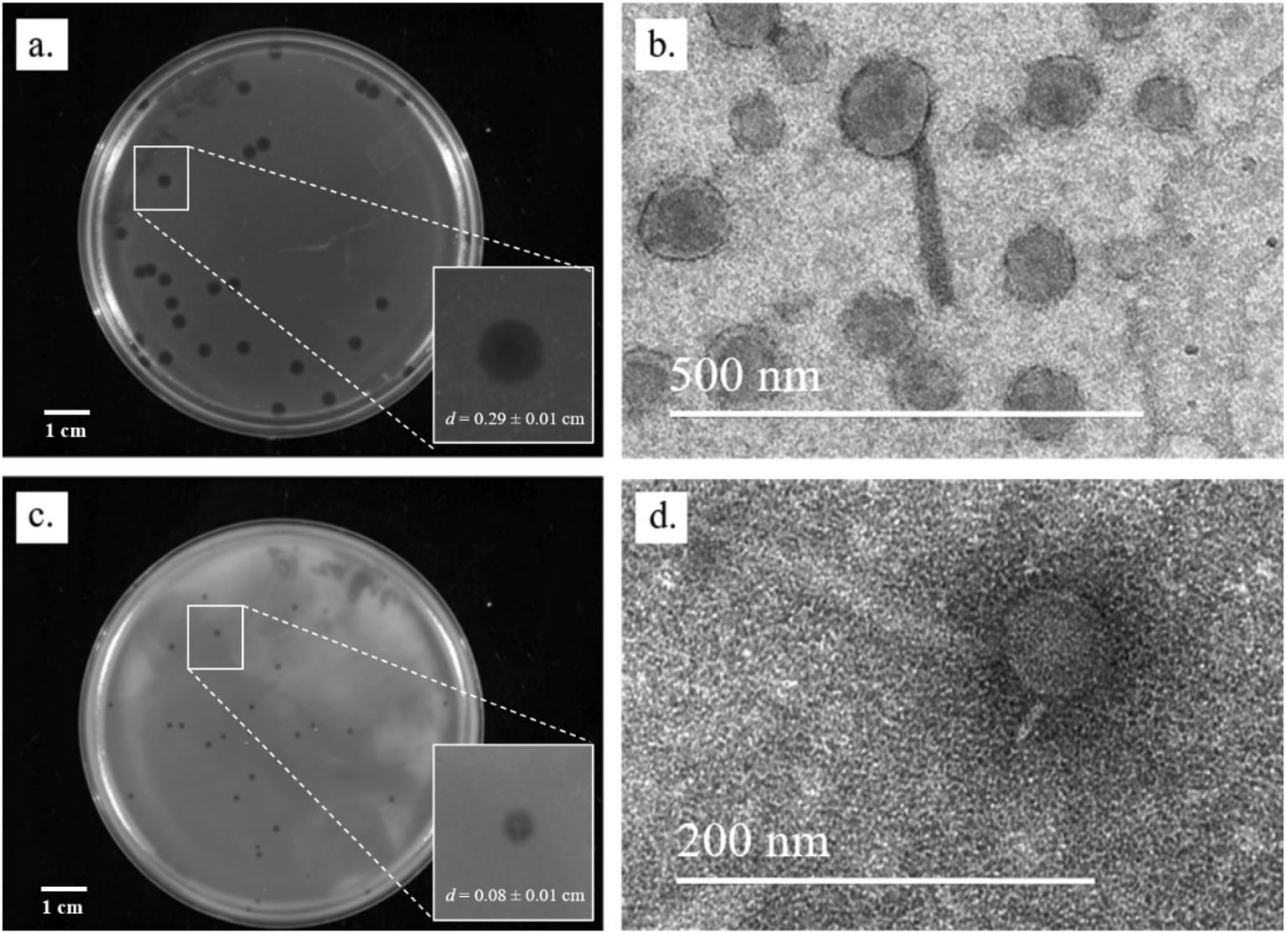
Plaque morphology and Transmission electron micrographs of purified phages, (**a**) plaques of ɸPh_PP01, (**b**) TEM image of ɸPh_PP01, (**c**) plaques of ɸPh_PP02, (**d**) TEM image of ɸPh_PP02.

ɸPh_PP01 produced large (0.29 ± 0.01 cm dia), circular, clear plaques, and on enrichment, the titre of the resultant lysate was 3.4 × 10^9^ PFU/mL. On the other hand, plaques of ɸPh_PP02 were small (0.08 ± 0.01 cm dia), circular, and achieved a titre of 2.3 × 10^9^ PFU/mL (Fig. 1a,c). TEM analysis showed variations with regard to dimensions; the diameter of head of ɸPh_PP01 is 29 nm, with a tail length of 67 nm, while ɸPh_PP02 showed 22 nm and 72 nm, respectively (Fig. 1b,d). These are tailed phages belonging to the class of C*audoviricetes* as per the latest classification of the International Committee on Taxonomy of Viruses (ICTV) ^51^.

#### Tropism

Both the phages ɸPh_PP01 and ɸPh_PP02 showed a zone of lysis against *E. coli* ST155 apart from their isolation host *P. putida,* respectively, indicating limited polyvalency (Table 2).

**Table 2:** Host range profile (tropism) of *P. putida* phages, ɸPh_PP01 and ɸPh_PP02. ‘+’ and ‘–’ represent sensitivity and resistance.

Bacterial strains	Phage Isolates	
	ɸPh_PP01	ɸPh_PP02
*E. coli* ST155	+	+
*P. fluorescence*	–	–
*P. aeruginosa* PAO1	–	–
*K. pneumoniae*	–	–
*S. enterica*	–	–
*E. coli MDR*	–	–
*P. vulgaris*	–	–
*S. marcesens*	–	–
*A. baumannii*	–	–
*S. aureus*	–	–

#### Phage adsorption and one-step growth curve

**Figure 2:**
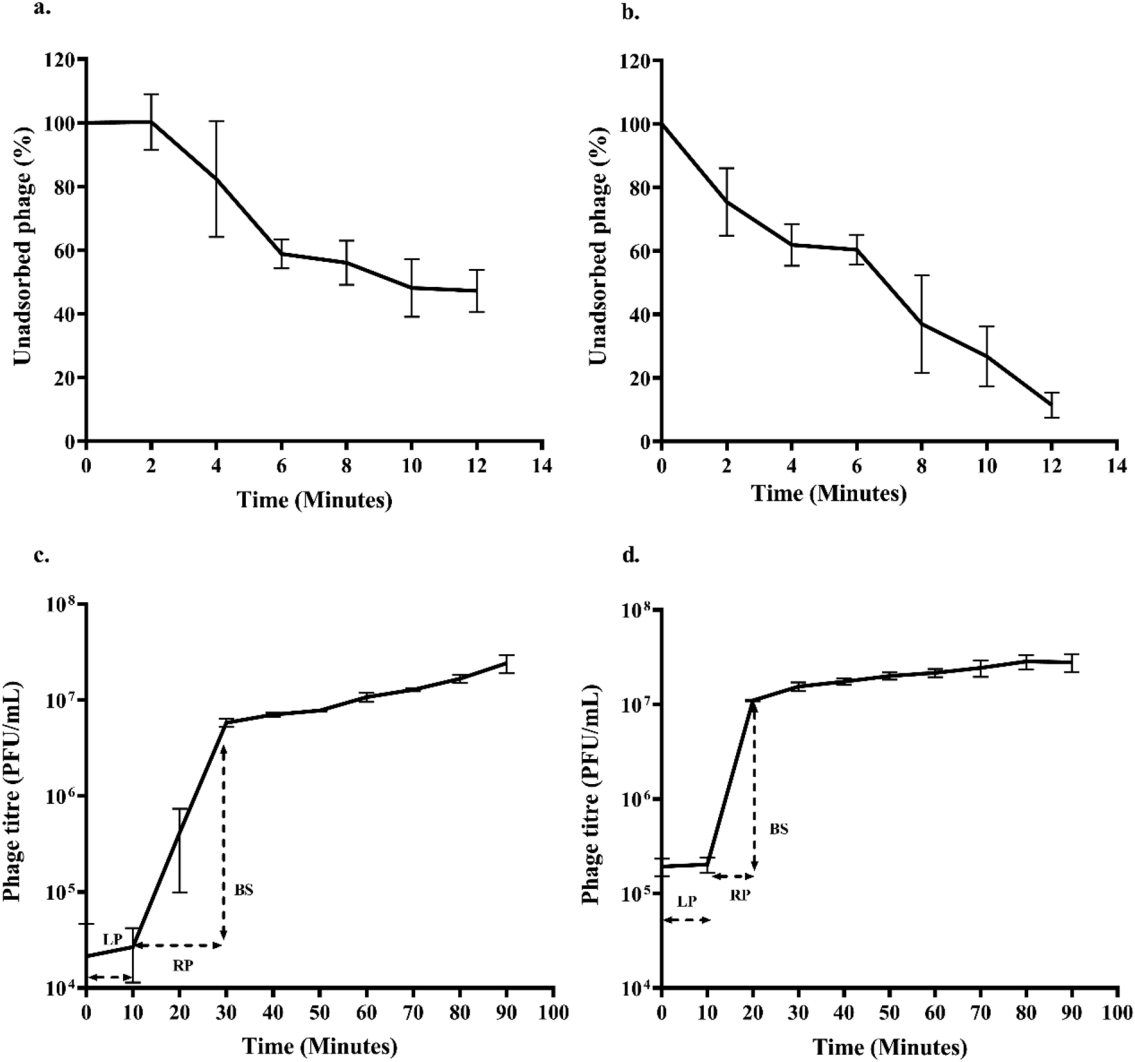
Adsorption rate and one-step growth curve kinetics of phage isolates on *P. putida.* Adsorption efficiency, (**a**) ɸPh_PP01, (**b**) ɸPh_PP02, one-step growth curve, (**c**) ɸPh_PP01, (**d**) ɸPh_PP02. LP-Latent period, RP-Rise period, BS-Burst size. The error bar represents the standard deviation.

The rate of adsorption of ɸPh_PP01 was close to 53% in 12 min, while ɸPh_PP02 showed a higher adsorption rate of 89% in the same time duration (Fig. 2a,b). On analysis of the one-step growth curve, the latent period of ɸPh_PP01 and ɸPh_PP02 was found to be approximately 10 min, while the rise period was 20 min for ɸPh_PP01 and 10 min for ɸPh_PP02, respectively. The burst size of the phages ɸPh_PP01 and ɸPh_PP02 were 507 ± 187 and 105 ± 7 virions per cell, as shown in (Fig. 2c,d).

#### Effect of pH, temperature, and chloroform on phage stability

**Figure 3:**
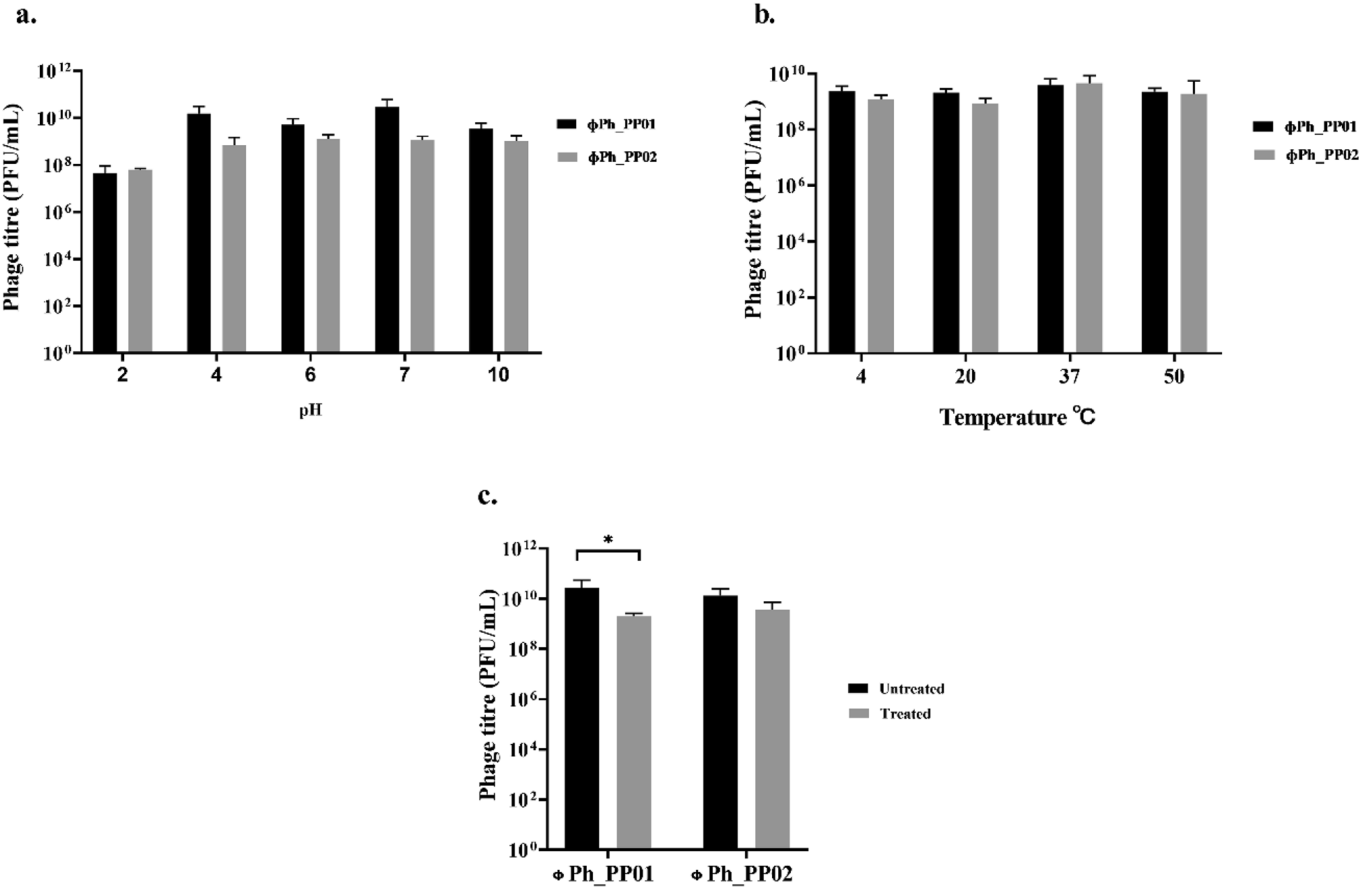
Stability studies, (**a**) pH, (**b**) Temperature, (**c**) 10% Chloroform.

The pH stability of the phages ɸPh_PP01 and ɸPh_PP02 are shown in (Fig. 3a). ɸPh_PP01 showed maximum stability at pH 4 through pH 7, minimum at pH 2 and with a slight reduction at pH 10. Whereas ɸPh_PP02 showed maximum stability at all pH ranging from 4 to 10 but with a reduced stability at pH 2. The results indicated that both phages ɸPh_PP01 and ɸPh_PP02 were stable at temperatures ranging from 4 to 50 ℃.(Fig. 3b). Moreover, the stability of phages in chloroform was analysed by treating them with 10% concentration. ɸPh_PP01 showed only a 1-log reduction in titre, while ɸPh_PP02 did not show any significant log reduction as indicated in Fig. 3c.

#### Restriction digestion

**Figure 4:**
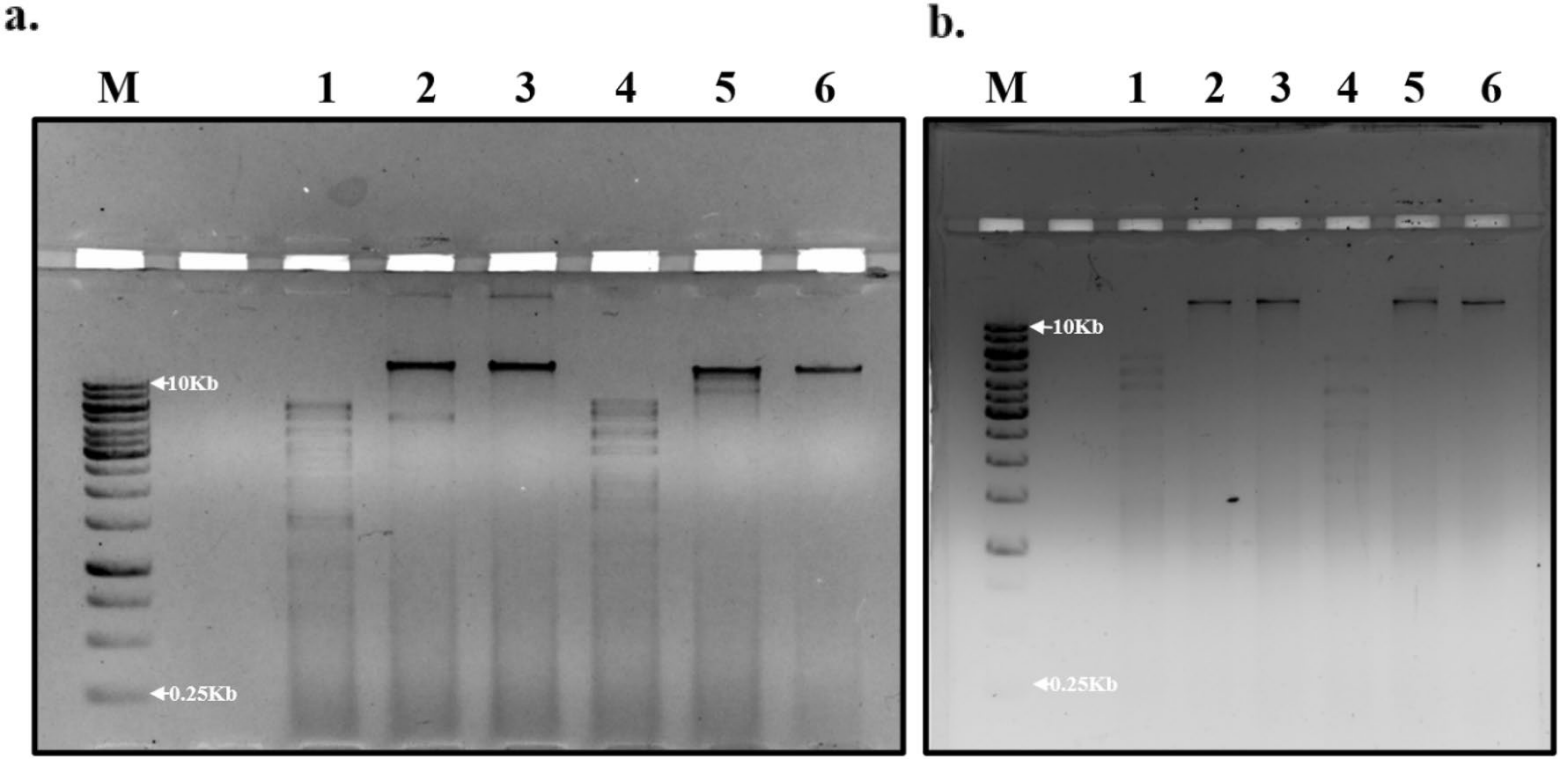
RFLP analysis of phages on 1% agarose gel stained with ethidium bromide, (**a**) ɸPh_PP01, (**b**) ɸPh_PP02. M- 1 Kb DNA ladder (GeneRuler, ThermoFisher Scientific), lane 1—*Eco*RI, lane 2—*Bam*HI, lane 3—*Hind*III, lane 4—*Hpa*I, lane 5—*Cla*I, lane 6—Uncut DNA. [Original figures are provided in the supplementary file, Figs. S1 and S2].

The very digestion indicated that the genomes are double-stranded DNA, and varying restriction profiles suggested compositional differences. Phage ɸPh_PP01 was sensitive to all restriction enzymes except *Hind*III, whereas ɸPh_PP02 was sensitive only to *Eco*RI and *Hpa*I and resistant to the rest (Fig. 4a,b).

### In vitro bacterial killing assay

**Figure 5:**
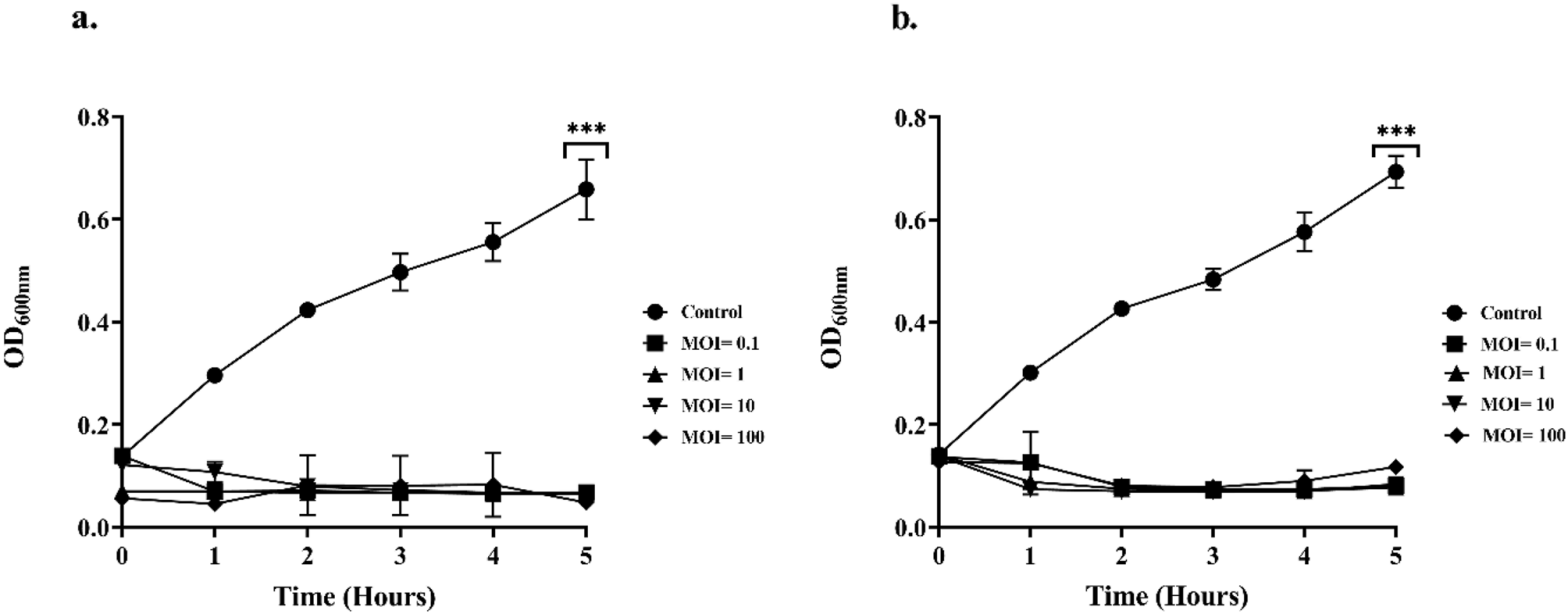
Bacteriolytic activity of phages against *P. putida* at different MOI. (**a**) ɸPh_PP01, (**b**) ɸPh_PP02. There is a significant reduction in bacterial growth after phage treatment at all MOI, *p* < 0.001.

In spite of the different doses applied, both phages showed intense lytic activity when compared to the control (without phages). Initially, there was a gradual rise in OD_600_ after a period of 1 h for bacteria infected at MOI 1 and MOI 100, but growth declined rapidly after that. A significant reduction in growth was observed at all four MOI’s for both phages over a period of 5 h. (Fig. 5a,b).

#### Phage resistant mutants

The frequency of development of mutants following phage treatment at MOI 10 was higher for ɸPh_PP02, while the BIMs for ɸPh_PP01 were comparatively lower even after 24 h. The resistance to phages was finally confirmed by the absence of plaques on the bacterial lawn (Table 3).

**Table 3:** Bacteriophage insensitive mutant frequency of *P. putida* at an MOI of 10. SD represents standard deviations of the three replicates done independently.

Phage	BIM frequency	
	CFU/mL/24 h	SD
ɸPh_PP01	1.11E-07	1.92E-08
ɸPh_PP02	1.04E-05	3.62E-07

#### Antibiotic sensitivity profile

The antibiotic sensitivity pattern of *P. putida* indicated that it is sensitive to amikacin, but showed intermediate sensitivity to ceftazidime, aztreonam, imipenem, and ciprofloxacin. It is resistant to ticarcillin which belongs to β-lactam class of antibiotics (Supplementary file, Table S1).

#### Prophage induction

**Figure 6:**
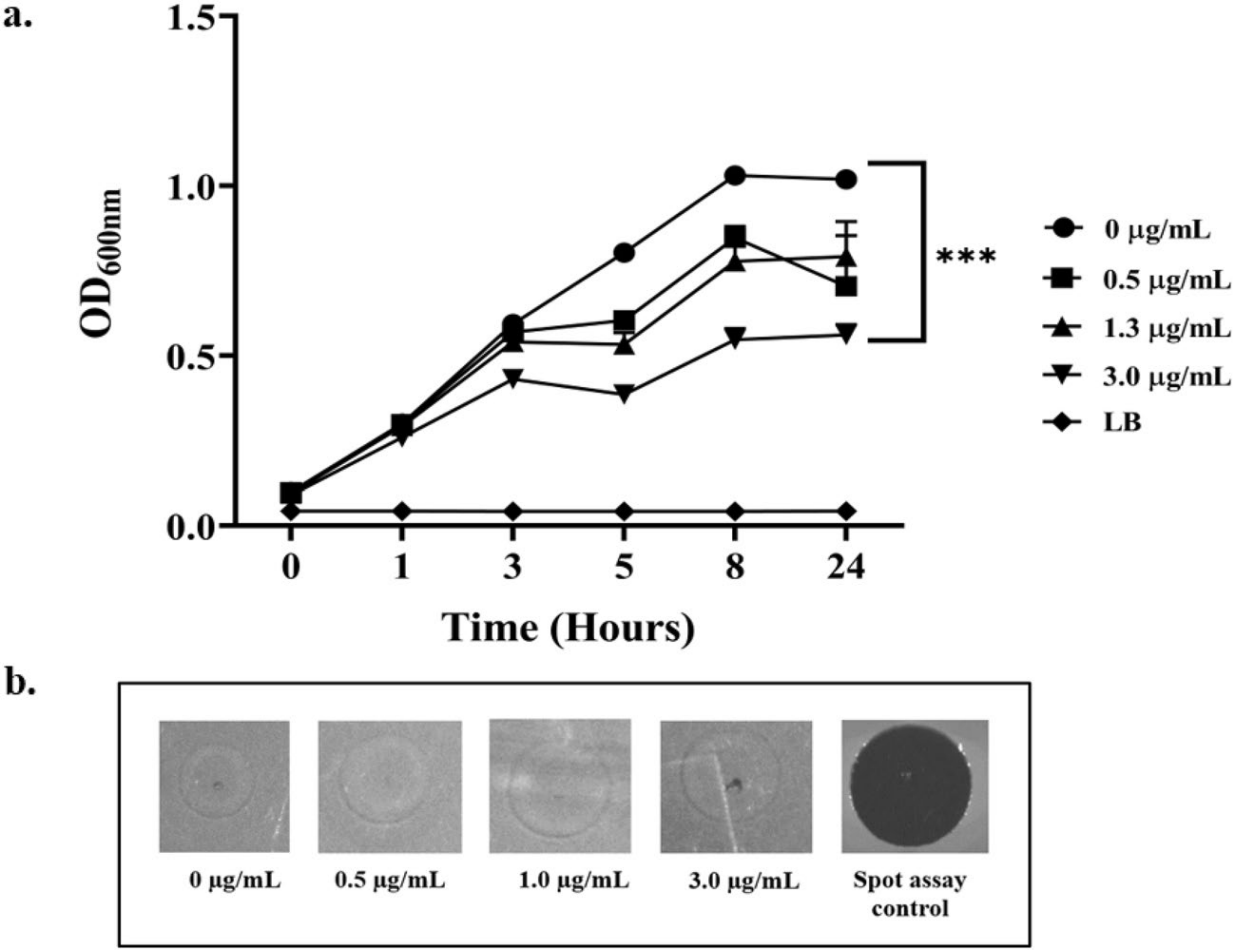
Induction of *P. putida* with mitomycin C to detect the presence of prophages. (**a**) Prophage induction of *P. putida* at different concentrations of mitomycin C for a time period of 24 h showing significant reduction in bacterial turbidity, *p* < 0.001. (**b**) Spot assay of different concentrations of cell-free lysate post 24 h treatment shows lack of lysis against *P. putida,* indicating the absence of active prophages.

The growth profiles revealed that there is a rapid reduction in the growth of the bacteria with the increase in mitomycin C concentration when compared to the control without mitomycin C. In addition, mitomycin C induction clearly indicated the absence of prophages as they showed no visible plaques (Fig. 6a,b).

### Phage activity in methanethiol reduction in synthetic sewage

**Figure 7:**
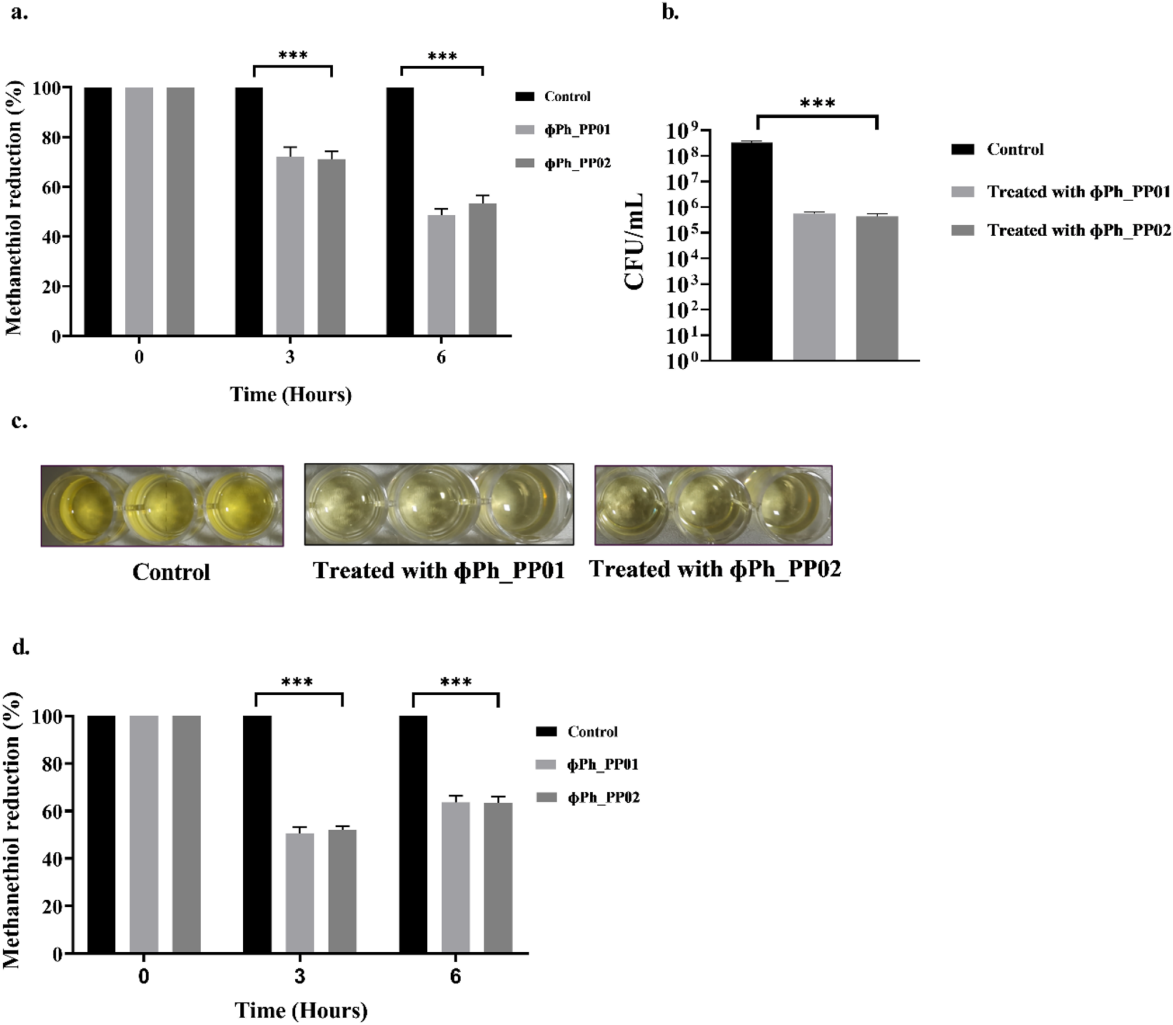
Quantification of methanethiol based on Ellman’s assay. (**a**) Reduction in biogenic methanethiol post 6 h treatment using phages ɸPh_PP01 and ɸPh_PP02, *p* < 0.001. (**b**) bacterial cell count reduction post 6 h treatment using phages ɸPh_PP01 and ɸPh_PP02, *p* < 0.001. (**c**) Image showing a reduction in methanethiol. (**d**) Effect of ɸPh_PP01 and ɸPh_PP02 on methanethiol biogenesis in *P. putida*-spiked sewage sample, *p* < 0.001.

Phage activity in the reduction of methanethiol was moderate in the initial hours (~ 3 h). But, a significant reduction of 52% and 47% were observed for both the phages, ɸPh_PP01 and ɸPh_PP02 post 6 h treatment when compared with the non-phage control, *p* < 0.001 (Fig. 7a). This observation was further supported by the visual differences in colour formation by TNB, which corresponds to the concentration of methanethiol produced by *P. putida* that decreased over time (Fig. 7c). Additionally, a 3-log reduction of *P. putida* was seen after phage treatment when compared to the control (synthetic sewage with *P. putida* and without phages) (Fig. 7b).

### Effect of phage treatment in sewage

The phage efficacy in reducing biogenic methanethiol in sewage (parameters of which are represented in supplementary Table S2) was significant in the initial 3 h with a reduction of 49% and 48% for ɸPh_PP01 and ɸPh_PP02, respectively, when compared with the non-phage control. But at 6 h, efficiency reduced to 36% with both the phages *p* < 0.001 (Fig. 7d).

## Discussion

The establishment of POC demanded the characterisation of phages, ɸPh_PP01 and ɸPh_PP02, and a methanethiol-producing target organism, *P. putida*. Most of the features employed for the purpose (viz. morphology, burst size, adsorption efficiency etc. for phages, and antibiotic sensitivity profile, BIM, and prophage induction for target bacteria) were chosen considering their relevance in wastewater settings. Both the phages, for example, displayed clear and discrete plaques (of differential sizes). This, when read along with their short latent period of 10 min in the one-step growth curve, indicates their lytic nature^52,53^. Wastewater applications prefer lytic over temperate phages due to several advantages^54^.

Polyvalent phages are best suited for environmental applications since it can infect different types of hosts, unlike its monovalent counterpart^55^. ɸPh_PP01 and ɸPh_PP02 showed limited polyvalency and were specific towards *P. putida*, their isolation host, and *E. coli* ST155. The latter is an established production host that can harbour multiple polyvalent phages, hence bypassing the need to involve pathogenic hosts for their production^56^. High polyvalency is advantageous, because effective malodour mitigation can be carried out with a formulation of limited phages than through a complex cocktail targeting the concerned bacterial population. In spite of its limitation, ɸPh_PP01 and ɸPh_PP02 were suitable for establishing a POC involving a single bacterial target.

The phages also exhibited differences in adsorption efficiency, rise period and burst size. High burst size is another ideal feature for application in wastewater due to its positive influence on MOI, and, in turn, on better reduction of target organisms. ɸPh_PP01 exhibited a longer rise period and a larger burst size when compared to ɸPh_PP02, which in turn showed a shorter rise period and smaller burst size, a correlation that is concordant with a previous study^57^. Moreover, phages with a higher adsorption rate will have a shorter lysis time ^58^ which is also observed in the current study, which will have positive implications on the duration of the treatment process and efficiency.

Phage activity varies depending on factors like bacterial cell density, pH, temperature, organic matter, etc^59^. The stability of the chosen phages at pH ranging from 2 to 10 indicates that they can be used in wastewater where pH fluctuates due to various factors. Approximately 75% of the world's global wastewater temperature falls within the range of 6.9–34.4 °C throughout the year^60^. Therefore, the thermal stability of phages is considered a major parameter for environmental applications, and this study proves both the phages are thermotolerant, withstanding 50 °C with insignificant reduction in titre. Isolated phages were found to be tolerant to 10% chloroform without any log-fold decrease of phage titre, which is contrary to literature where 2 log phage inactivation happens within 5 min. Lipid-containing phages are more vulnerable to chloroform treatment^61^.

MOI is not the sole determinant of phage dosing but is nevertheless an important factor. It is generally considered that infection proportionally increases with increasing MOI^62,63^. In spite of this, MOI is just one of several parameters that influence the likelihood of phages finding their targets and establishing an infection, so it is important to experiment on real samples in order to gain more insight into this process^59^. Over a period of 5 h, the phages used in establishing POC reduced *P. putida* significantly while operating at different MOIs.

Several factors contribute to the evolution of phage resistance in bacterial hosts, including sustained exposure to phages and acquisition of fitness advantages^64^. High frequency in the development of bacteriophage-insensitive mutants in *P. putida* is counterproductive in the application of bacteriophages, for example, in malodour mitigation. The development of BIMs will render the infection process ineffective since the introduced phages no longer identify the host. This scenario necessitates the need for repeated development of phage preparation. The defence mechanisms evolved by bacteria are diverse, and the use of phage cocktails or polyvalent phages targeting different mechanisms can further reduce the development of resistance^65^.

The lack of mitomycin C induction showed that *P. putida* does not harbour any intact prophages, which is an ideal feature of the host employed in POC establishment. Mitomycin C induction involves triggering the cell’s SOS mechanism as well as the *rec* A, thereby inducing prophages^66^ . Prophage induction in *P. putida* with mitomycin C gave a reduction in turbidity at OD_600_ since it is toxic to the cell, and a spot test of the lysate with host did not show any plaque formation, strongly indicating the absence of active prophages*.* Additionally, as a strain descriptor, antibiotic sensitivity profiling was also done, though it did not have an implication on the POC establishment.

Synthetic sewage augmented with *P. putida* was used as the sample to establish the POC. In synthetic sewage, phages could achieve a concomitant reduction of 52% and 47% within 6 h with a reduction in bacterial count of 3 log. However, studies in synthetic sewage do not represent the heterogeneous microbial communities present in the real scenario. Hence, the experiment was extended to wastewater spiked with *P. putida* in which maximum reduction in methanethiol was achieved in 3 h, but at 6 h, the efficiency was reduced, which might be due to the non-specific binding of phages to decoys and reduced phage adsorption and infection efficacy^67^. It is imperative to monitor the impact of phages on wastewater, which is a diverse and dynamic biological system. Spectrophotometry-based assays have low sensitivity and are, therefore, not ideal. Hence GC, based techniques need to be adopted for effective monitoring. Iteration regarding time and MOI can improve the reduction further. Identifying the percentage reduction needed to reduce methanethiol below the olfactory threshold will require further research.

Few studies, including this, have demonstrated the potential of phages in mitigating biogenic malodour in wastewater settings. Field applications in decentralised wastewater systems require understanding the microbiome composition of wastewater. In a large-scale system, treating industrial wastewater showed inverse correlations between phage concentrations and bacterial hosts^68^. A better understanding of the wastewater microbiome is crucial for effective phage treatment for several reasons, such as target identification, resistance monitoring, phage selection, dosage optimization, etc. The rationale involves targeting keystone species and a subset of heterogenous microbial populations implicated in malodour generation. Phage cocktail formulations targeting these organisms that produce different biogenic malodourants, such as hydrogen sulphide, methanethiol, ammonia, and organic acids, need to be constituted to bring about aspirational malodour reduction. It is essential that phage dissemination systems, such as LBS (Lytics Broadcasting System) and BAR-LBS (Bacteriophage Amplification Reactor-LBS), along with appropriate production hosts, be in place to ensure efficient translation^69^. Expanding these systems with phage cocktail preparations, multiple malodourants can be targeted in wastewater efficiently and economically. The advanced odour abatement technologies are highly effective for removing bacteria, but most developing countries, like India, are unable to deploy these systems widely where decentralised sanitation is mostly preferred due to population density and urbanisation. Phage based mitigation of malodour causing organisms bypasses the physical and chemical conventional odour treatment strategies in an eco-friendly manner. This POC establishment study has proven the potential of phages as an alternative biological treatment method for malodour mitigation, paving the way for applications in full-scale systems.

### Supplementary Information

Supplementary Information.

## Data Availability

All data generated or analysed during this study are included in this published article and its supplementary information files.
