# Supplementary material for: Mitigation of biogenic methanethiol using bacteriophages in synthetic wastewater augmented with Pseudomonas putida
Source: Sci Rep. 2023 Nov 9;13:19480. doi: 10.1038/s41598-023-46938-8 (PMC10636157; doi:10.1038/s41598-023-46938-8)
Supplement: Supplementary file 1 — Supplementary Information. [file 41598_2023_46938_MOESM1_ESM.pdf]

Mitigation of biogenic methanethiol using bacteriophages in synthetic wastewater augmented with *Pseudomonas putida*

Niti Sarat, Amrita Salim, Sanjay Pal*, Suja Subhash, Megha Prasad, Bipin G. Nair, Ajith Madhavan*

School of Biotechnology, Amrita Vishwa Vidyapeetham, Kerala-690525, India

*Dr. Ajith Madhavan: ajithm@am.amrita.edu

*Dr. Sanjay Pal: sanjaypal@am.amrita.edu

**Antibiotics class**	**Antibiotics**	**Sensitivity profile of *P. putida***	**Zone of inhibition in *mm***	**EUCAST reference zone of inhibition in mm**
β-lactam	Ticarcillin	R	6	≤ 18
3rd generation Cephalosporin	Ceftazidime	I	25	17-49
Monobactem	Aztreonam	I	35	18-49
Carbapenem	Imipenem	I	30	21-49
Aminoglycoside	Amikacin	S	24	≥ 15
Fluoroquinolones	Ciprofloxacin	I	33	26-49

Table S1: Antibiotic sensitivity profile of *P. putida* against different classes of antibiotics. Symbol S- sensitive, R- resistance, I- intermediate. The zone of inhibition (mm) was measured and interpreted as per the EUCAST guidelines.

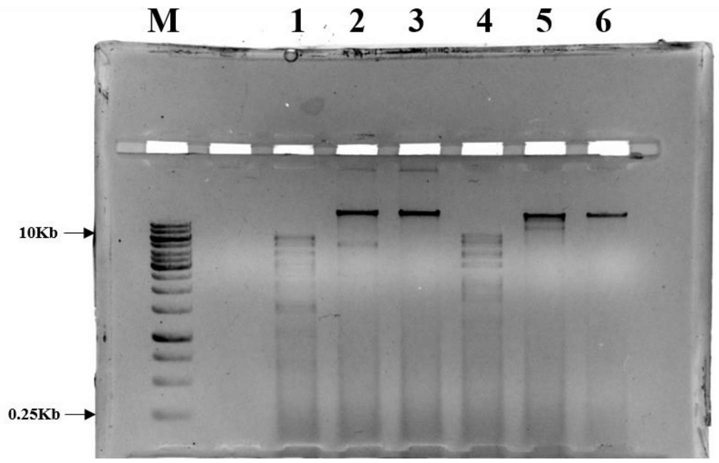

Figure S1: Restriction digestion profile of ϕPh_PP01 genome on 1% agarose gel and stained with ethidium bromide. M- 1Kb DNA ladder, lane 1- *Eco*RI, lane 2- *Bam*HI, lane 3- *Hind*III, lane 4- *Hpa*I, lane 5- *Cla*I, lane 6- uncut phage DNA.

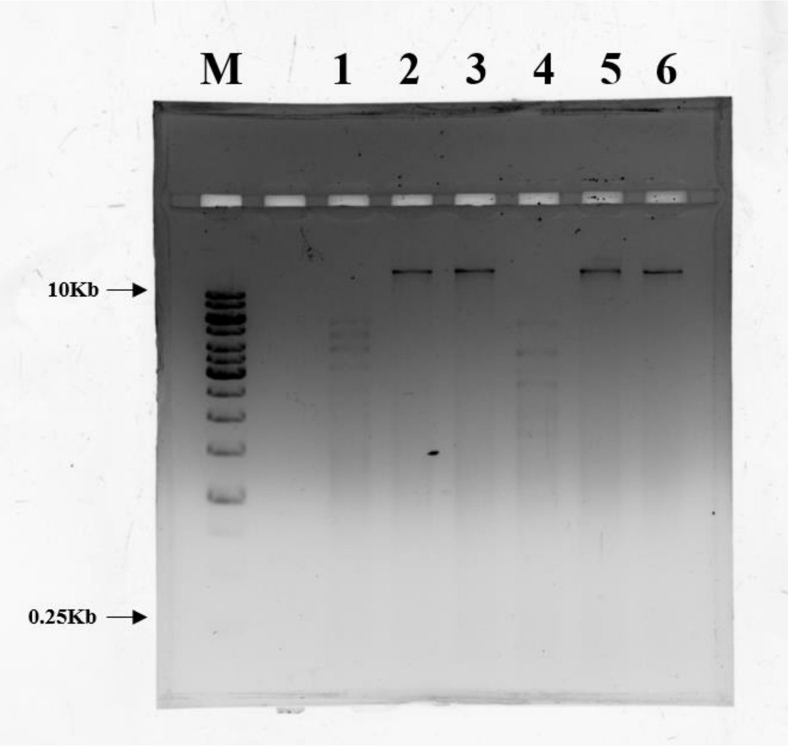

Figure S2: Restriction digestion profile of ϕPh_PP02 genome on a 1% agarose gel stained with ethidium bromide. M- 1Kb DNA ladder, lane 1- *Eco*RI, lane 2- *Bam*HI, lane 3- *Hind*III, lane 4- *Hpa*I, lane 5- *Cla*I, lane 6- uncut phage DNA.

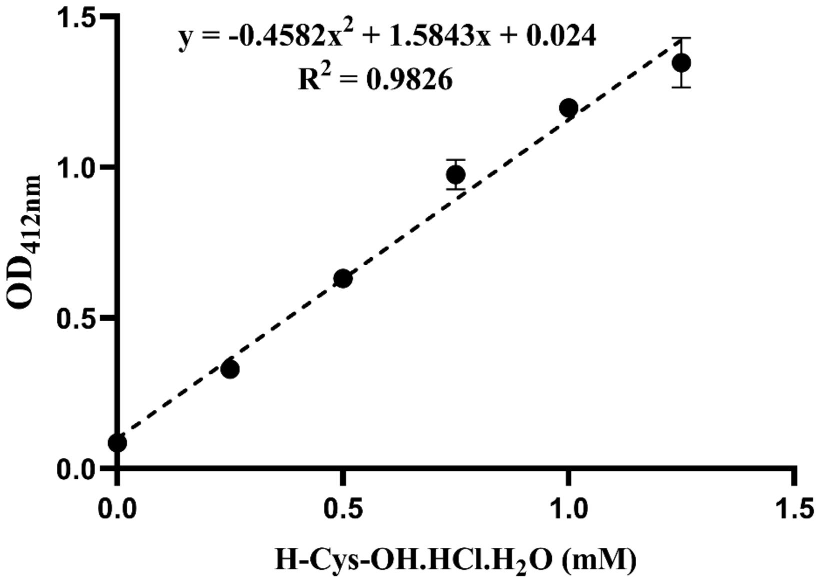

Figure S3: Ellman's assay. Standard graph generated with 0 - 1.25 mM concentration of H-Cys-OH.HCl.H_2O (cysteine hydrochloride monohydrate).

**Parameters**	**Result**
pH	6.90
Total Suspended Solids(TSS)	58.0 mg/L
Biochemical Oxygen Demand(BOD)	56.0 mg/L
Chemical Oxygen Demand(COD)	144 mg/L

Table S2: Parameters of sewage used in POC establishment.
